# Emergency intubation using a light wand in patients with facial trauma

**DOI:** 10.4103/0974-2700.44685

**Published:** 2009

**Authors:** Sandeep Sahu, Apurva Agarwal, Avaneet Rana, Indu Lata

**Affiliations:** Department of Anaesthesiology, GSVM Medical College, Kanpur, UP, India; 1 Department of Anaesthesiology, VMMC and Safdarjung Hospital, New Delhi, India

**Keywords:** Difficult airway management, faciomaxillary injuries, light wand

## Abstract

Airway management in the operating room is the responsibility of anesthesiologists, although a variety of personnel may be responsible for airway management outside the operating room. Emergency department physicians are prominently involved in airway management in the emergency room both independently and with anesthesiologists. Airway management in trauma patients remains the domain of anesthesiologists. An 18-year old male patient was brought to our emergency room after an alleged history of suicidal attempt with gunshot under the chin. He was scheduled to undergo emergency tracheotomy, debridement, and closure of facial laceration under general anaesthesia, presenting a challenge for. He had to undergo emergency tracheotomy, debridement, and closure of facial lacerations under general anesthesia. The injuries made the patient's airway management a complex issue. We present the use of the light wand to manage the difficult airway of this patient with complex facial trauma.

## INTRODUCTION

Generally, faciomaxillary injuries are due to trauma, road traffic accidents but in our case it was a suicidal attempt by gunshot. So, it is a rare case managed by light wand to be reported till now.

## CASE REPORT

An eighteen-year-old male patient was brought to our emergency department five hours after an alleged history of suicidal attempt with gunshot under the chin. He was presented with a burst open face and no recognizable structures on the face, except for the eyes. He was uncooperative and drowsy. His arterial blood pressure was 80/60 mm Hg and pulse rate was 124/min. His respiratory rate was 26/min and was slightly distressed. He was unable to lie supine as the shredded structures tended to fall back; causing airway obstruction and blood trickled into the oropharynx causing him considerable distress.

**Figure 1:**
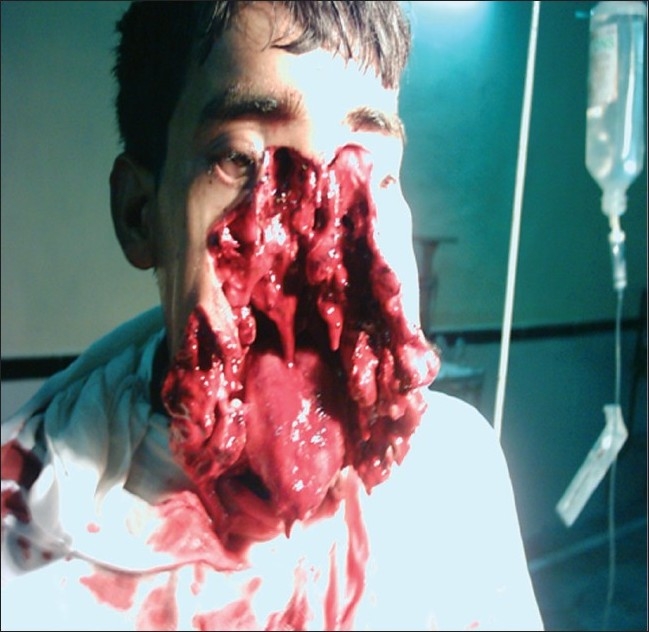
Photo at time of admission in emergency department, showing extent of injuries

After initial resuscitation in emergency department with intravenous fluids (colloid and crystalloids) and blood transfusion patient was stabilized and suctioning of the blood from the upper airway, a closer examination of the face was done. There was comminuted fracture of mandible, maxilla, and nasal bones. Tongue, hard palate, and nasal structures were not recognizable [Figure 1]. Three loose teeth were seen embedded in the lower half of the face. The eyes were spared and his vision was unimpaired. Cerebrospinal fluid leak could not be made out because of the presence of blood. The expired gases had an exit near the root of nasal structure, which could be made out by the movement of cotton strands and no foreign body was present. The neck was not injured. Nervous system examination, as far as could be elicited, was normal with no cranial nerve damage or sensory and motor weakness. On auscultation, the breath sounds were normal with no added sounds, suggesting no aspiration of blood into trachea. Rest of the systemic examination was also normal. Chest radiograph was normal and no foreign body was present. He was scheduled to undergo emergency tracheostomy for airway management, debridement, and closure of facial laceration under general anaesthesia.[1]

### Anaesthetic management

The patient was shifted to the operating room in the sitting position. We planned for tracheal intubation under sedation using light wand, in anticipation of a difficult airway.[2,3] Pulse oximeter, ECG leads, non-invasive blood pressure monitor etc. were connected. Difficult airway cart was kept ready as patient was resuscitated and stabilized vitals (BP 110\84 mm Hg, PR 96\min, SPO2 96%). Injection Propofol 60 mg in sedation doses was administered intravenously slowly to enable the patient to lie supine. Airway was cleared by suctioning and holding forward of the fractured bones. The loose teeth were easily pulled out lest they should get dislodged. After adequate preoxygenation with a mask held close to the face we tried to perform direct laryngoscopy, but to our dismay, the blade could not be maneuvered once it was introduced into the oral cavity. Thereafter, we went for intubation using light wand.[4] The operating room lights were dimmed and a well-lubricated, 8.0 mm endotracheal tube mounted light wand, which had been pre-shaped in the hockey stick manner was inserted in the oral cavity The usual technique of light wand intubation was used and the patient was easily intubated in the first attempt. The correct placement of the endotracheal tube was confirmed by chest auscultation. The entire process took thirty seconds. The tube was fixed securely with the help of a bandage. The anaesthesia was maintained with 40:60 O2: N2O, inj. Vecuronium bromide and halothane. The surgeons proceeded with the tracheostomy. Despite meticulous dissection, they had difficulty in locating the trachea. The light wand was introduced into the endotracheal tube and the transillumination directed the surgeons towards the exact location.[5] As the surgeon was about to incise the trachea, the endotracheal tube cuff was deflated and the light wand was removed. When the trachea was adequately incised, the orally placed endotracheal tube was slowly pulled out and a 7.5 mm ID cuffed tracheostomy tube was inserted. The breathing circuit was connected to the tracheotomy tube. Facial laceration was debrided and closed. The entire surgical procedure took 1.5 hours. Giving reversal reversed the residual effect of neuromuscular blockade. After ensuring an adequate tidal volume and when the patient was following commands patient was shifted in the postoperative room. Oxygen inhalation was continued in the postoperative period. The patient was monitored till 24 hours for any complications. Then patient was referred for a CT scan of head, face, and neck. Three-dimensional CT scan of face showed complex comminuted fracture of face involving bilateral maxilla (anterior, lateral, medial wall, and alveolar process), hard palate, orbital wall, nasal bones, septum, and roof of nose and left sided base of pterygoid plate with opacification of bilateral maxillary, ethmoidal, and sphenoid sinuses. Zygomatic bones, eyeball, and optic nerve were normal. CT scan of head and neck were normal.

**Figure 2:**
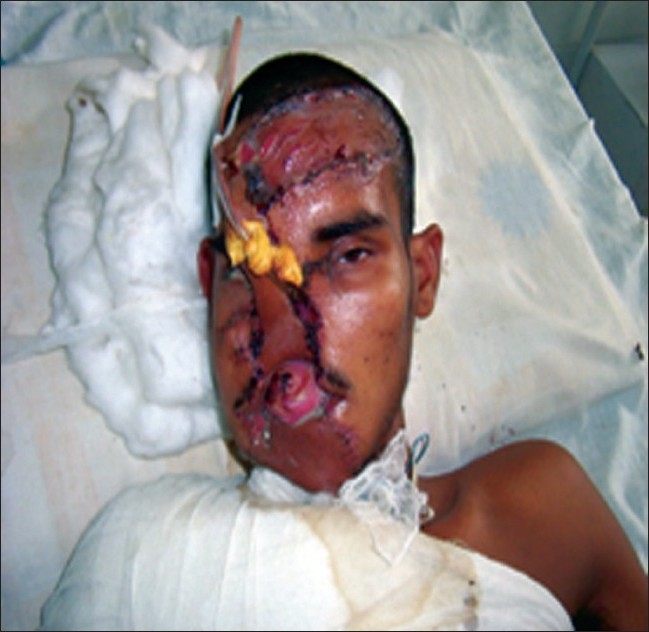
After reconstructive surgeries and airway management with light wand

He was posted for definitive repair of face three days later when the edema of the bilateral ramus and body of mandible, left coronoid process of mandible face had subsided. The surgical procedure took 3.5 hours. The patient continues to do well waiting for bone grafting and prosthesis implant [Figure 2].

## DISCUSSION

The primary concern in a patient with facial injuries is to secure the airway.[2] This may be rendered challenging because of distorted anatomy, haemorrhage, oedema, and presence of foreign bodies. There are various supraglottic and fiberoptic devices are available to manage such difficult airway like LMA, Glidescope, fiberoptic larangoscopes that are helpful to manage such conditions. Besides this it can be managed with retrograde or submental intubation[6,7] and nasotracheal intubation that needed expertise. Tracheotomy is an efficient means, especially for long-term maintenance of airway.[7] This is usually accomplished under local anaesthesia. In our case, the patient was unable to lie supine because of airway obstruction caused by loose lacerated tissues and blood. Hence, tracheal intubation was necessary to prevent aspiration of foreign material into the trachea and to enable tracheotomy in supine position. The most appropriate means of endotracheal intubation in patients with facial trauma has been debated. Fiberoptic intubation without neuromuscular blockade, though an outstanding means in other situations, cannot be used in presence of blood in the upper airway as it impairs the visibility,[8] such as in our case. Awake intubation can be an ideal alternative to fiberoptic intubation in difficult airways management. Nasotracheal intubation is frequently used for airway management during maxillofacial surgery. Complications such as haemorrhage occur more frequently with this route of intubation than with the orotracheal route.[8] Blind nasal intubation was not performed fearing dislodgement of the endotracheal tube into the cranium and because of entirely shattered nasal structures.[9] Direct laryngoscopy was attempted but was unsuccessful probably because the support provided by soft tissues and mandible was absent. Attempts using an intubating laryngeal mask airway and SLIPA, supraglottic airway devices did not succeed. As there was no availability of fiberoptic larangosope and other fiberoptic assisted devices, airway was finally managed by the most appropriate technique left was to use the light wand.

Light wand is a safe, effective and rapid technique for oral as well as nasal intubation in patients with difficult airway and in patients in which minimal neck movements are desired, such as in patients with cervical spine injuries. It can be used in both awake and apnoeic patients. It does not require the mandibular support as required in direct laryngoscopy. The light wand can be used in patients with limited mouth opening and abnormal airway. In our case, the soft tissues of the neck and cervical spine were unaffected, which helped us achieve a good transillumination, permitted cricoid's pressure, and allowed neck movements.

The light wand can prove to be an unique device in locating the trachea while performing tracheostomy. The transillumination provided is very bright and localized which cannot be achieved by any other device. The intensity of the glow seems to be unaffected by the presence of an endotracheal tube in situ. This can be particularly useful in the scenario where trachea cannot be identified during tracheostomy owing to displacement of trachea. This may be seen in patients with blunt or penetrating injuries to the neck, as well as in some cases of neck mass. Hung *et al*. has described light guided retrograde intubation in patients with cervical spine injuries but the light wand was not used to facilitate the retrograde catheterization of trachea.

To conclude, the light wand is a very useful device for tracheal intubation in patients with facial trauma. Life saving tracheal intubations can be performed smoothly and rapidly. This makes it an ideal device to be used routinely in our emergency rooms.
